# A Rare Case of Monophasic Synovial Sarcoma of Thoracic Vertebra

**DOI:** 10.1155/2018/2313927

**Published:** 2018-11-18

**Authors:** Sana Najib, Tahmina Saleem, Ali Nadhim, Shuvendu Sen

**Affiliations:** Raritan Bay Medical Center, Perth Amboy, NJ, USA

## Abstract

Synovial sarcoma of spine is an extremely rare malignancy with poor prognosis. It is often metastatic at the time of presentation. Its relative rarity and histological resemblance to other tumors make it diagnostically challenging, requiring the need of immunohistochemistry and cytogenetics for definite diagnosis. Surgery is the mainstay of therapy with adjunct chemotherapy, although survival rates are very low.

## 1. Introduction

Synovial sarcoma is a rare, soft tissue tumor comprising only 5% of all soft tissue sarcomas [1, 2]. It predominantly arises in the deep tissues of extremities, with only 5% arising from the axial skeleton [3]. It usually affects young adults and has a male predominance [4]. It has a very poor prognosis with metastatic disease, with survival rates of less than 2% over 5 years.

## 2. Case Presentation

**Figure 1:**
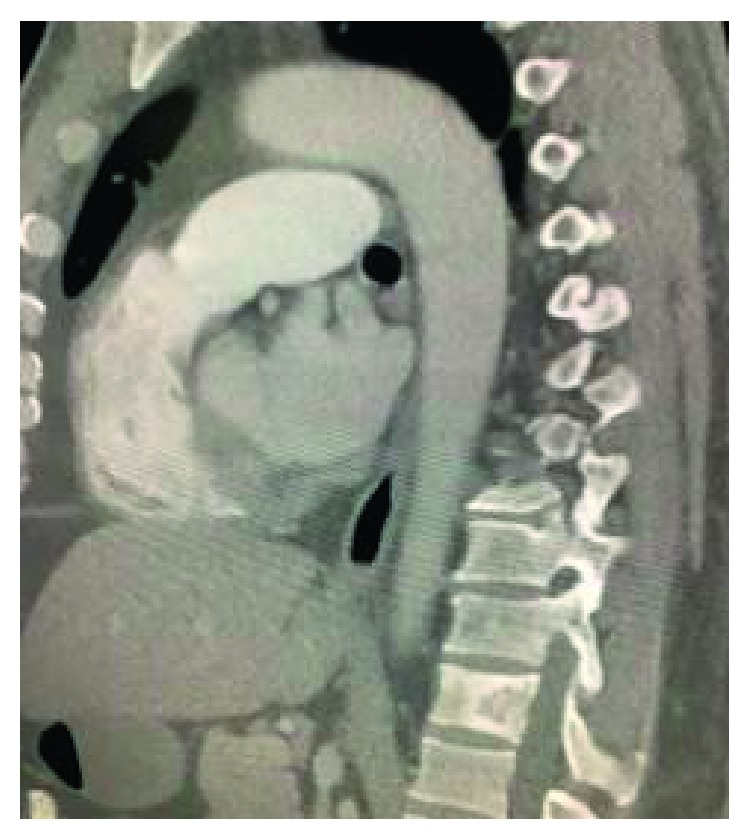
Sclerotic and lytic lesions at t12.

**Figure 2:**
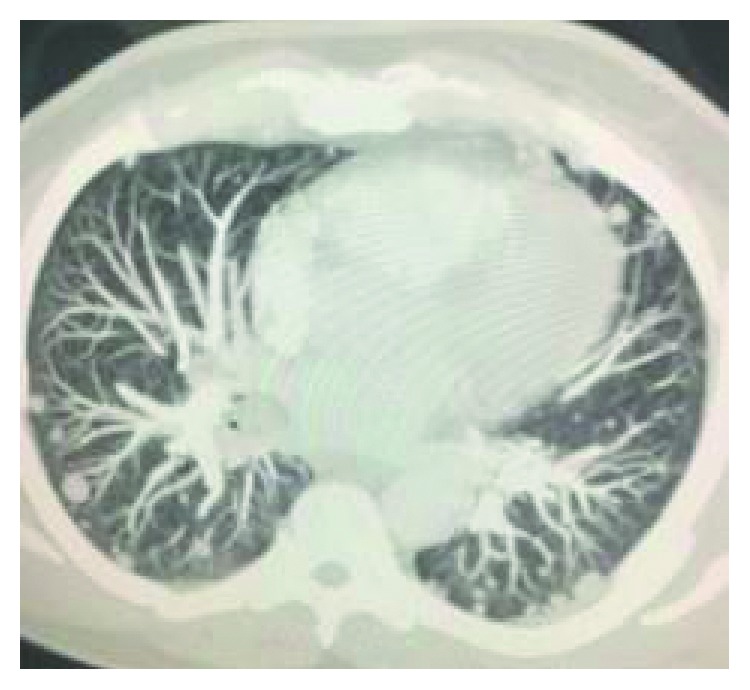
Bilateral pulmonary nodules.

**Figure 3:**
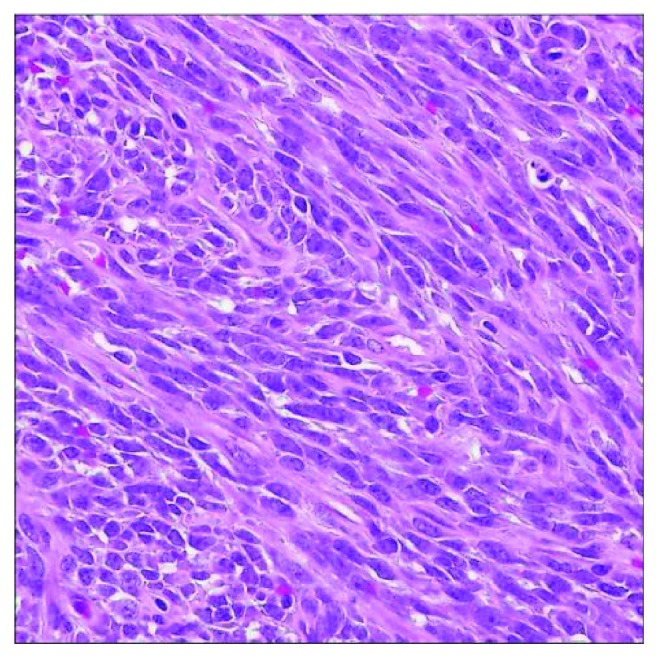
Histology with H&E staining and high-power magnification showing spindle cells suggestive of monophasic variant of synovial sarcoma.

A 44-year-old male with no significant past medical history presented to us with the complaint of lower back pain radiating to both groins for the last 2 months. Vitals at presentation were BP of 120/70 mm Hg, pulse of 82, RR of 18/minute, and T of 98.9°F. Physical exam and lab work were unremarkable. CT scan showed a sclerotic and lytic lesion in T12 (Figure 1). Further investigation with CT scan of chest revealed multiple bilateral pulmonary nodules along with mediastinal and hilar lymphadenopathy suggestive of a metastatic malignant process (Figure 2). CT-guided biopsy of a pulmonary nodule showed malignant neoplasm with spindle cells (Figure 3). Immunohistochemical stains showed tumor cells positive for Bcl-2, vimentin, EMA, CD56, CD99, and TLE-1 and negative for CD34, CD10, P63, cytokeratin, S100, desmin, and synaptophysin. Interphase FISH was performed and was positive for a rearrangement involving SYT gene (18q11) consistent with a synovial sarcoma. Biopsy of the T2 lesion showed exactly the same IHC and FISH findings. The whole-body CT scan for extrathoracic disease was negative. The patient was referred to another facility for further management.

## 3. Discussion

**Figure 4:**
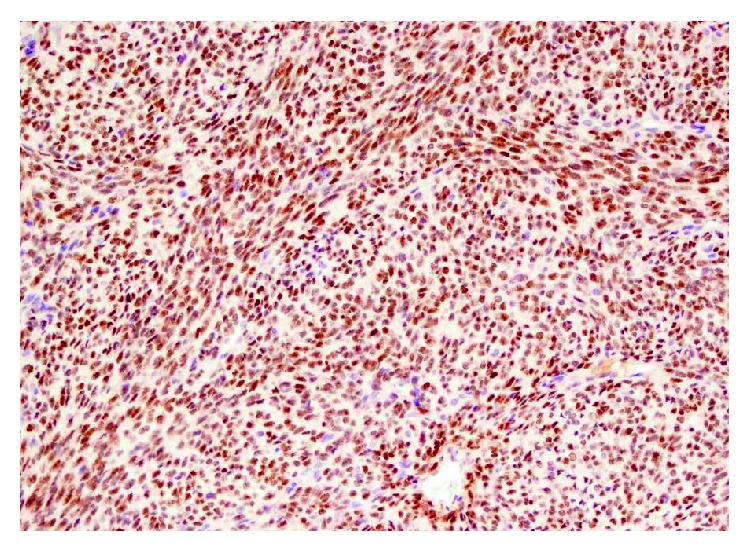
TLE-1 immunohistochemistry.

**Figure 5:**
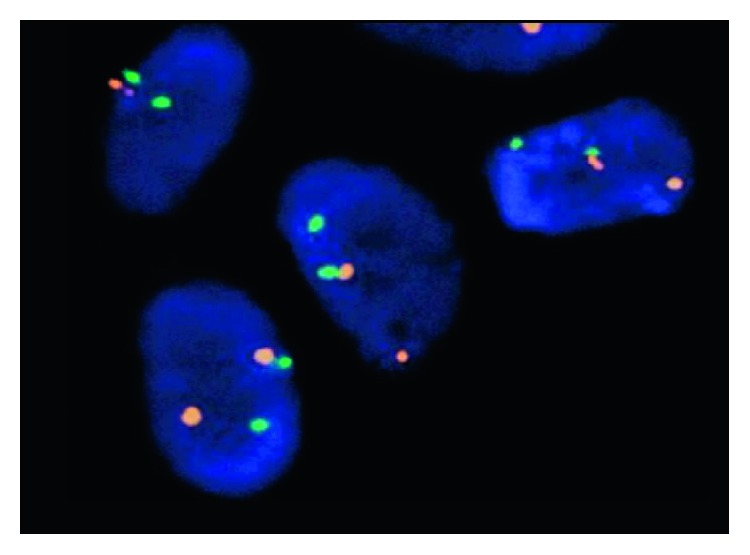
FISH showing SYT split.

Synovial sarcoma of the spine is an extremely rare tumor. It has multiple subtypes, out of which monophasic (spindle cell carcinoma) has close histological resemblance to other tumors including fibrosarcoma, leiomyosarcoma, and spindle cell variant of squamous cell carcinoma that are relatively more common. Histopathology is insufficient to make diagnosis, and immunohistochemistry is mandatory to rule out other neoplasms on differentials [5]. In the past, IHC has been used as the sole method of diagnosis but review of the literature has shown cases, where IHC was inconclusive showing synovial sarcoma to be positive for markers such as S-100 making diagnosis challenging (Figure 4). Advances in cytogenetics have revealed that the definitive diagnosis of synovial sarcoma can be made only by evidence of a translocation t(X : 18) characterized by fusion of the SYT gene (Figure 5) on chromosome 18 with SSX genes on X chromosome resulting in a chimeric protein [5]. Recently, this gene product is the target for the development of newer antineoplastic agents for synovial sarcoma that are under clinical trials (Figure 5).

## 4. Conclusion

This case report raises the awareness about synovial sarcoma, a rare malignancy with extremely poor prognosis. Refractory back pain in a young patient should be investigated properly for any possibility of a malignant process. This malignancy is diagnostically challenging, requiring cytogenetic translocation for definitive diagnosis [5]. Although prognosis is poor, favorable outcomes have been seen in cases with early detection and appropriate surgical management with adjunctive chemotherapy and radiotherapy [6].
